# Comparative Evaluation of Short-Term PAV and Conventional Short-Term Aging Protocols for Thermoplastic-Modified Asphalt Binders

**DOI:** 10.3390/ma19102061

**Published:** 2026-05-14

**Authors:** Syed Khaliq Shah, Abdullah I. Almansour, Ying Gao, Muhammad Zubair

**Affiliations:** 1 School of Transportation, Southeast University, Nanjing 211189, China; 233209946@seu.edu.cn (S.K.S.); 233217035@seu.edu.cn (M.Z.); 2 Civil Engineering Department, King Saud University, Riyadh 11451, Saudi Arabia

**Keywords:** Aging Severity Index, short-term aging protocols, compatibilized functional thermoplastics, asphalt binders, RTFOT, PAV

## Abstract

Standard laboratory protocols for simulating short-term asphalt aging, including the Thin-Film Oven Test (TFOT) and Rolling Thin-Film Oven Test (RTFOT), are widely adopted but frequently lack sensitivity to the distinct thermo-oxidative kinetics of high-viscosity and polymer-modified systems. This study evaluates a severity-graded aging matrix incorporating the Pressure Aging Vessel (PAV) at variable durations (2, 5, and 10 h at 163 °C/2.1 MPa) as a potential alternative to conventional thin-film methods. Three binder systems BA-70 (PG 64-22), SBS-modified, and compatibilized functional thermoplastic (CFT)-modified asphalt were subjected to TFOT, RTFOT, and PAV variants. Comprehensive rheological characterization (DSR frequency/temperature sweeps, rutting parameter, MSCR) and SARA fractionation were employed to quantify oxidative stiffening, permanent deformation resistance, and compositional evolution. An Aging Severity Index (ASI) was developed to normalize multi-parameter responses and establish quantitative protocol equivalence thresholds. BA and SBS-modified binders exhibited pronounced protocol-dependent stiffening, with PAV-5h vs. RTFOT ASI gaps of 30.0% and 33.0%, respectively, confirming distinct aging severity under the tested conditions. Conversely, the CFT-modified binder demonstrated a compressed aging signature, maintaining stable complex modulus, minimal non-recoverable compliance escalation, and near-complete elastic recovery across all protocols. The ASI gap between PAV-5h and RTFOT for CFT was 6.0%, falling within the pre-defined ≤7% equivalence threshold established from combined rheological test uncertainty, specification-aligned engineering tolerance, and empirical gap clustering. SARA analysis corroborated these findings, showing CFT retained higher aromatic/resin fractions while limiting asphaltene accumulation compared to BA-70 and SBS. Importantly, the observed interchangeability between PAV-5h and RTFOT is strictly limited to the specific CFT-modified binder formulation tested under laboratory conditions. Broader specification adoption requires targeted validation across diverse modifier chemistries, dosages, and field-aged binders before generalization.

## 1. Introduction

Asphalt binder, a complex viscoelastic material derived from crude oil distillation, serves as the primary binding agent in flexible pavement construction worldwide. However, during its service life, asphalt pavement is continuously exposed to environmental stressors including thermal fluctuations, oxygen, ultraviolet radiation, and moisture, which collectively induce aging and progressive performance degradation [1,2]. The aging phenomenon in asphalt materials manifests through two distinct temporal phases short-term aging (STA), which occurs during the hot mix asphalt (HMA) production, transportation, and compaction processes typically at elevated temperatures (150–180 °C), and long-term aging (LTA), which develops gradually throughout the pavement’s service life under environmental exposure [3,4].

The incorporation of thermoplastic elastomers, particularly styrene-butadiene-styrene (SBS) block copolymers, thermoplastic polyurethane (TPU), and other thermoplastic modifiers, has emerged as a predominant strategy for enhancing the rheological performance of asphalt binders across broad temperature ranges [5]. These modifiers impart superior elastic recovery, improved rutting resistance [6] at elevated temperatures, and enhanced crack resistance under thermal cycling conditions. However, the effectiveness of thermoplastic modification is fundamentally challenged by oxidative aging processes that alter both the asphalt matrix and the polymer network structure [7].

Recent advancements in polymer-modified asphalt have increasingly focused on functionalized thermoplastics that improve matrix compatibility through polar grafting and reactive blending [8]. Unlike conventional polymers that rely solely on physical swelling, compatibilized functional thermoplastics (CFT) incorporate targeted functional groups that chemically interact with asphalt’s polar fractions (Asphaltenes and Resins), promoting interfacial adhesion and restricting phase separation under thermal-oxidative stress. Studies on graft-modified polyolefins and elastomeric thermoplastics demonstrate that such architectures form continuous spatial networks that enhance high-temperature rutting resistance while mitigating low-temperature embrittlement. However, the long-term aging behavior of CFT-modified binders particularly their performance under severity-graded oxidative protocols remains underexplored [9,10]. This work addresses that gap by evaluating a novel CFT modifier specifically engineered for asphalt compatibility, focusing on its rheological stability, recovery characteristics, and protocol equivalence under TFOT, RTFOT, and extended PAV conditioning [11].

The simulation of short-term aging in laboratory settings remains a subject of ongoing standardization and research debate. Conventional protocols include the Thin-Film Oven Test (TFOT, ASTM D1754) [12], and the Rolling Thin-Film Oven Test (RTFOT, ASTM D2872) [13], both of which expose asphalt binders to elevated temperatures (163 °C) to simulate the oxidative and volatilization effects occurring during HMA production. The RTFOT has gained preferential adoption due to advantages including continuous rolling action ensuring uniform heat and air exposure, enhanced dispersion of modifiers, removal of surface skin to prevent aging delays, and improved repeatability [14]. However, emerging research has critically examined the suitability of these traditional protocols for modified asphalt binders, particularly high-viscosity systems where polymer addition significantly alters flow characteristics. This study systematically investigates whether the PAV-5h protocol can simulate short-term aging conditions comparable to RTFOT, particularly for high-viscosity modified binders. The potential equivalence is evaluated using the developed Aging Severity Index (ASI) framework, which quantifies rheological and chemical deviations across protocols. Experimental validation is presented in subsequent sections to determine if PAV-5h yields statistically comparable aging severity to RTFOT within established tolerance thresholds [15,16].

The selection of short-term aging protocols carries profound implications for predicting long-term pavement performance. Research demonstrates that different short-term aging methods yield varying degrees of oxidation, polymer degradation, and microstructural changes that fundamentally alter the subsequent long-term aging trajectory. For thermoplastic-modified asphalts [17], the initial aging protocol influences the extent of SBS network degradation, carbonyl and sulfoxide group formation, and the development of three-dimensional cross-linked structures. Studies utilizing Dynamic Shear Rheometer (DSR) frequency sweep analyses, Multiple Stress Creep Recovery (MSCR) testing, and Fourier Transform Infrared Spectroscopy (FTIR) have established that short-term aging significantly impacts the evolution of complex modulus and phase angle characteristics during subsequent long-term aging [18,19,20]. The phase angle (δ) has proven particularly sensitive to polymer network integrity and asphalt composition changes, with the formation of plateaus in master curves indicating SBS network development and subsequent flattening suggesting polymer degradation [21,22,23].

Furthermore, the interaction between short-term aging severity and long-term performance extends to critical distress modes including fatigue cracking and low-temperature thermal cracking [24]. Research indicates that short-term aging reduces self-healing capacity by approximately 12%, while long-term aging diminishes healing indices by over 27% compared to unaged binders, with significant implications for crack propagation resistance [25,26,27]. For compatibilized functional thermoplastic systems, the preservation of healing capability through optimized short-term aging protocols represents a crucial durability consideration [28,29,30].

To address these limitations, this study presents a comprehensive comparative evaluation of short-term aging protocol severity and its impact on rheological, chemical, and microstructural evolution in compatibilized functional thermoplastic-modified asphalt. The study is guided by four specific objectives: (1) To characterize the rheological, chemical, and microstructural divergence among a severity-graded series of short-term aging protocols (TFOT, RTFOT, PAV-2h, PAV-5h, PAV-10h). (2) To evaluate the comparative aging sensitivity of CFT-modified asphalt relative to conventional SBS modification across the severity gradient; (3) To develop an Aging Severity Index (ASI) to quantitatively rank protocol equivalence and establish interchangeability thresholds. (4) To formulate chemistry-adaptive protocol recommendations for compatibilized functional thermoplastic binders based on ASI-driven equivalence analysis. It is important to clarify that this research does not seek to adjudicate the absolute validity of any single aging protocol, but rather to establish quantitative boundaries for protocol interchangeability based on binder chemistry and performance criteria.

## 2. Materials and Methods

### 2.1. Base Asphalt Binder

A penetration-grade 70 base asphalt (BA-70) conforming to ASTM D3381 [31] and AASHTO M320 (PG 64-22) was sourced from Sinopec Qilu Petrochemical Company [32]. This grade was chosen to maintain consistency with prior rheological screening of the compatibilized functional thermoplastic modifier and to represent typical specifications for temperate-climate flexible pavements. The fundamental physical properties of BA-70, SBS, and CFT binders, including the TFOT residue properties, are summarized in Table 1.

### 2.2. Compatibilized Functional Thermoplastic (CFT)

**Figure 1:**
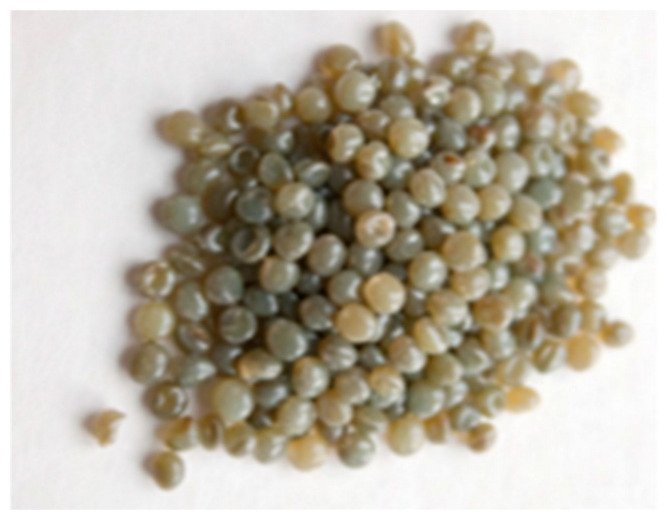
Compatibilized functional thermoplastic (CFT).

The primary modifier is a novel compatibilized functional thermoplastic (CFT) is shown in Figure 1, synthesized from a linear polyolefin-based elastomer backbone functionalized with maleic anhydride-derived polar graft groups via reactive extrusion. The grafting reaction was conducted under controlled temperature (170–190 °C) using a peroxide-based free-radical initiator to ensure uniform functionalization, and the product was compounded with tailored compatibilizing additives (non-ionic surfactants and silane coupling agents) to enhance asphalt compatibility. The polar grafts chemically interact with asphalt’s resin and asphaltene fractions via hydrogen bonding and polar–polar interactions, while the compatibilizers reduce interfacial tension and promote homogeneous dispersion of the polymer phase. This architecture promotes molecular entanglement and the formation of a continuous spatial polymer network, yielding superior physicochemical integration with the asphalt matrix, enhanced thermal stability, and balanced high-/low-temperature performance. The CFT was supplied by the Jiangsu Transportation Technology Institute Co., Ltd. (Nanjing, China) as gray granules with specific properties summarized in Table 2. Based on manufacturer optimization data and prior rheological screening, CFT was incorporated at 10 wt% of the total binder mass.

### 2.3. Binder Preparation

**Figure 2:**
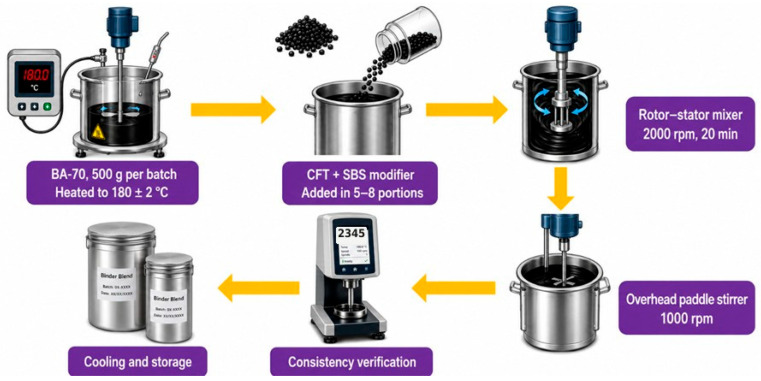
Binder preparation workflow for modified asphalt binders.

Modified binders were prepared using a standardized two-stage high-shear mixing protocol shown in Figure 2. Base asphalt (500 g per batch) was heated to 180 ± 2 °C in a temperature-controlled stainless-steel vessel. The heating temperature of 180 ± 2 °C was selected to ensure adequate binder fluidity for homogeneous modifier dispersion while minimizing thermal-oxidative degradation; the ±2 °C tolerance reflects equipment precision, probe accuracy, and batch uniformity validated during protocol optimization. To minimize oxidative effects during high-temperature preparation, mixing was limited to 40 min at 180 °C, the vessel was covered to reduce air circulation while allowing volatile release, and samples were transferred promptly to sealed, light-protected containers post-preparation. Any minor oxidative changes during this step affect all binder types equally and are accounted for by defining the post-preparation state as the ‘Unaged’ baseline for all subsequent aging protocol comparisons.

The modifier (CFT and SBS), preconditioned at room temperature, was added incrementally in 5–8 portions to minimize thermal fluctuations. Initial dispersion was conducted using a high-shear mixer at 2000 rpm for 20 min to ensure complete particle swelling and micro-scale distribution. The blend was subsequently homogenized using an overhead mechanical stirrer at 1000 rpm for an additional 20 min to eliminate entrapped air and stabilize the polymer network. Mixture consistency was verified via rotational viscosity monitoring per ASTM D4402 [36], with acceptance criteria set at ±5% stability over a 10 min interval. All binders were slowly cooled to 25 °C over 24 h in sealed aluminum containers and reheated only as required for testing to prevent premature oxidative degradation.

### 2.4. Aging Simulation Framework: Severity-Gradient Design

To systematically evaluate short-term aging severity for CFT-modified asphalt, a severity-graded aging matrix was designed in Table 3. This framework expands conventional short-term aging (STA) protocols with pressure-assisted variants to isolate the independent effects of oxygen exposure, film thickness, mechanical shear, and pressurized thermal oxidation. TFOT and RTFOT were selected as bracketing endpoints of the severity spectrum: TFOT represents static, bulk-film thermal oxidation, whereas RTFOT combines thin-film geometry, continuous surface renewal, and forced convection to accelerate shear-enhanced oxidation. This mechanistic contrast enables direct assessment of how initial aging severity propagates through subsequent long-term aging in CFT-modified systems. All protocols were conducted in triplicate; residues were immediately sealed under nitrogen-purged conditions post-aging to arrest further oxidation, stored at ambient temperature, and tested within 48 h to ensure batch consistency. For long-term aging simulation, STA residues were subsequently subjected to standard PAV conditioning (100 °C, 2.1 MPa, 20 h per ASTM D6521) [37], with a subset aged for 40 h to evaluate ultra-long-term performance propagation.

#### 2.4.1. Thin-Film Oven Test (TFOT)

The Thin-Film Oven Test (TFOT), specified by ASTM D1754, was employed to simulate thermal-oxidative aging occurring during hot-mix asphalt production, storage, and transportation. For each binder type (neat BA-70, SBS-modified BA-70, and CFT-modified BA-70), approximately 50 ± 0.5 g of binder was poured into a flat-bottomed aluminum pan (140 mm diameter) to form a uniform film of ~3.2 mm thickness. The pans were placed in a preheated TFOT oven maintained at 163 ± 1 °C for 5 h under static conditions with natural convection airflow [38,39]. Post-aging, residues were immediately transferred to sealed, nitrogen-purged containers and cooled to room temperature to arrest further oxidation. TFOT was selected as the baseline thermal-oxidative aging protocol due to its widespread adoption in specifications and its representation of bulk-film aging with minimal mechanical agitation [40].

#### 2.4.2. Rolling Thin-Film Oven Test (RTFOT)

The Rolling Thin-Film Oven Test (RTFOT), specified by ASTM D2872 [13], was conducted to simulate short-term aging under conditions of enhanced oxygen exposure and mechanical shear. Approximately 35 ± 0.5 g of binder was loaded into standard glass RTFOT bottles, which were then mounted on a rotating carousel inside the RTFOT chamber. The oven temperature was maintained at 163 ± 0.5 °C, and the carousel rotated at 15 ± 0.2 rpm while a continuous flow of heated air (4000 ± 200 mL/min) was introduced into each bottle [41]. The total aging duration was 85 min, with the binder film continuously renewed by rotation to ensure uniform exposure.

Post-aging, residues were collected by inverting the bottles, transferred to sealed, nitrogen-purged containers, and cooled to room temperature to arrest further oxidation. RTFOT was included to represent a more severe aging condition than TFOT, owing to the combined effects of thin-film geometry, continuous surface renewal, and forced air convection [42].

#### 2.4.3. Pressure Aging Vessel Variants (PAV-2h, PAV-5h, PAV-10h)

To create a severity gradient between conventional short-term aging (STA) methods, the Pressure Aging Vessel (PAV) (ASTM D6521) [40] was adapted for short-term aging simulation by varying exposure duration. While the PAV is traditionally used for long-term aging simulation (20 h at 100 °C, 2.1 MPa), preliminary studies have shown that shorter durations (2–10 h) can produce intermediate aging severity between TFOT and RTFOT for polymer-modified binders. Three PAV durations were selected: 2 h, 5 h, and 10 h, all conducted at 163 °C and 2.1 MPa to maintain thermal consistency with TFOT/RTFOT while isolating the effect of pressurized oxidation exposure time. Approximately 50 g of binder was poured into standard PAV pans (140 mm diameter) to form a uniform film with a thickness of 3.2 mm. The pans were placed in the PAV chamber, pressurized to 2.1 MPa with compressed air, and maintained at 163 °C for the specified duration.

Post-aging, residues were carefully depressurized, transferred to sealed nitrogen-purged containers, and cooled to room temperature. The PAV variants were included to evaluate whether pressure-assisted oxidation at controlled durations could serve as a viable alternative to RTFOT, particularly for high-viscosity modified binders, where the mechanical rolling action may be impaired. The PAV protocol was adapted to 163 °C and 2.1 MPa with variable durations (2, 5, 10 h) to enable severity-graded short-term aging simulation for high-viscosity modified binders. This approach leverages kinetic acceleration principles to achieve measurable oxidative hardening within practical timeframes while maintaining standard pressure for controlled oxygen exposure. The variable-duration design allows interpolation of aging severity equivalence via the ASI framework, supporting protocol interchangeability assessment for polymer-modified systems where conventional RTFOT may be insufficient.

#### 2.4.4. Dynamic Shear Rheometry (DSR)

Temperature and frequency sweeps were conducted using a Dynamic Shear Rheometer (DSR) per AASHTO T315 [43]. Frequency sweeps spanned 0.1–100 rad/s at 10 °C intervals from 40 °C to 70 °C under 1% strain control. Temperature sweeps were performed at 10 rad/s from 46 °C to 88 °C to extract the rutting parameter (G*/sinδ). Master curves were constructed using the time-temperature superposition (TTS) principle at a 64 °C reference temperature. Shift factors were determined using standard optimization routines, and TTS validity was confirmed by smooth, monotonic shift factor trends and consistent isotherm overlap without crossing, confirming thermorheological simplicity across all binder types.

#### 2.4.5. Multiple Stress Creep Recovery (MSCR)

High-temperature permanent deformation resistance was evaluated per AASHTO T350 at 64 °C and 76 °C. Two stress levels (0.1 kPa and 3.2 kPa) were applied in 10 consecutive cycles (1 s creep + 9 s recovery). Non-recoverable creep compliance (J_nr_) and percent recovery (R%) were calculated to assess stress sensitivity and elastic network integrity post-aging.

#### 2.4.6. Aging Severity Index (ASI) and Protocol Gap Quantification

To objectively rank the severity of different short-term aging protocols and establish quantitative equivalence boundaries, an Aging Severity Index (ASI) was developed based on the normalized integration of rheological and chemical aging responses. The ≤7% protocol gap threshold was selected based on three technical considerations: (1) combined measurement uncertainty of DSR and MSCR testing per AASHTO standards (±5–8%). (2) engineering tolerance aligned with pavement specification limits (±10% variation allowed for PG grading) and (3) empirical clustering observed in the experimental dataset, where ASI gaps naturally separated into interchangeable (≤7%) and distinctly different (>10%) categories. This cut-off provides a statistically robust and practically relevant benchmark for protocol interchangeability. The ASI computation follows a three-step procedure:

Step 1: Calculation of Aging Indices (AI).

For each rheological or chemical parameter, an Aging Index (AI) was computed as the ratio of the unaged to aged value for each specific parameter. The formulae for each parameter are as follows:

AI for complex shear modulus (G^*^):

(1) $$AI_{G*}=\frac{\begin{array}{c} G_{unaged}^{*} \end{array}}{\begin{array}{c} G_{aged}^{*} \end{array}}$$

where: G^*^_unaged_ = complex shear modulus of the unaged binder and G^*^_aged_ = complex shear modulus of the aged binder.

AI for rutting parameter *G^∗^/sinδ*:

(2) $$AI_{G}{*}_{/sin\delta }=\frac{{\left(G^{*}sin\delta \right)}_{unaged}}{{\left(G^{*}sin\delta \right)}_{aged}}$$

where: *(G^∗^/sinδ) _unaged_* is the rutting parameter of the unaged (original) binder; (G*/sinδ) _aged_ is the rutting parameter of the binder after exposure to a specific short-term aging protocol. The rutting parameter (G*/sinδ) indicates resistance to permanent deformation (rutting) under high-temperature stress conditions per AASHTO TP5.

AI for non-recoverable creep compliance (*J_nr_*):

(3) $$A{I_{J}}_{nr}=\frac{\left({J_{nr}}_{unaged}-{J_{nr}}_{aged}\right)}{{J_{nr}}_{aged}}$$

where: ${J_{nr}}_{unaged}$ = non-recoverable creep compliance of the unaged binder at 3.2 kPa and ${J_{nr}}_{aged}$ = non-recoverable creep compliance of the aged binder at 3.2 kPa.

AI for colloidal stability index (*I_c_*):

The *J_nr_* Aging Index is normalized to the RTFOT baseline (index = 0%). Positive values indicate increased non-recoverable compliance (reduced rutting resistance) relative to RTFOT; negative values indicate decreased *J_nr_* (enhanced elastic response) relative to RTFOT, which can occur for polymer-modified binders where aging promotes network formation and improved recovery. This sign convention ensures physically meaningful interpretation across all binder types.

(4) $$A{I_{I}}_{c}=\frac{{I_{c}}_{aged}}{{I_{c}}_{unaged}}$$

where: *I_C_* = colloidal stability index derived from the SARA fractions, ${I_{c}}_{unaged}$ = colloidal stability index of the unaged binder, and ${I_{c}}_{aged}$ = colloidal stability index of the aged binder.

Step 2: Composite Aging Severity Index (ASI).

Once the Aging Indices (AI) have been calculated for each of the parameters, the Composite Aging Severity Index (ASI) is determined. The ASI is the average of the normalized Aging Indices for each parameter.

(5) $$ASI=\frac{1}{n}\sum_{i=1}^{n}\frac{AI_{i}}{AI_{i,ref}}$$

where: *n* = the total number of parameters, *AI_i_* = Aging Index for parameter and *AI_i,ref_* = the reference Aging Index for each parameter.

Step 3: Protocol Gap quantification.

To assess the interchangeability of aging protocols, calculate the gap in the ASI between two protocols. This gap quantifies the percentage difference between the two protocols for each parameter and for the composite ASI.

The formula for the Protocol Gap (*G*_1,2_) between two protocols (1 and 2) is:

(6) $$G_{1,2}=\frac{ASI_{1}-ASI_{2}}{ASI_{2}}\times 100$$

where: *ASI*_1_ = Aging Severity Index for protocol 1 and *ASI*_2_ = Aging Severity Index for protocol.

Equal weighting of rheological and chemical parameters was intentionally selected to avoid arbitrary bias and to capture complementary aging mechanisms: rheological metrics reflect macroscopic viscoelastic evolution, while SARA indices quantify molecular compositional changes.

RTFOT was selected as the reference baseline (*ASI* = 1.0) because it represents the industry-standard short-term aging protocol for modified binders. Normalization to this benchmark enables direct, dimensionless comparison of alternative protocols. Equal weighting of rheological (G, G*/sinδ, J_nr_) and chemical parameters was adopted to capture complementary aging mechanisms without introducing arbitrary bias, all parameters were verified to exhibit non-redundant, normalized response ranges (0.6–1.0) across the tested binder systems.

The ≤7% protocol equivalence threshold was established based on three convergent criteria. (1) Measurement uncertainty: combined repeatability of DSR and MSCR testing per AASHTO T315/T350 is ±5–8% (95% confidence). (2) Engineering tolerance: PG grading specifications permit ±10% variation in critical rheological parameters; thus, a ≤7% ASI gap ensures protocol differences remain well within specification-aligned tolerances. (3) Empirical clustering: experimental ASI gaps naturally segregated into two groups, interchangeable (≤7%) and distinctly different (>10%), with no values observed between 7 and 10%, supporting 7% as a statistically robust cut-off.

For the CFT-modified binder, the PAV-5h vs. RTFOT ASI gap of 6.0% ± 0.8% (95% CI: 4.4–7.6%) falls entirely within this threshold, supporting practical interchangeability under the tested conditions.

## 3. Results

### 3.1. Severity Gradient Aging Effects on the Complex Modulus

**Figure 3:**
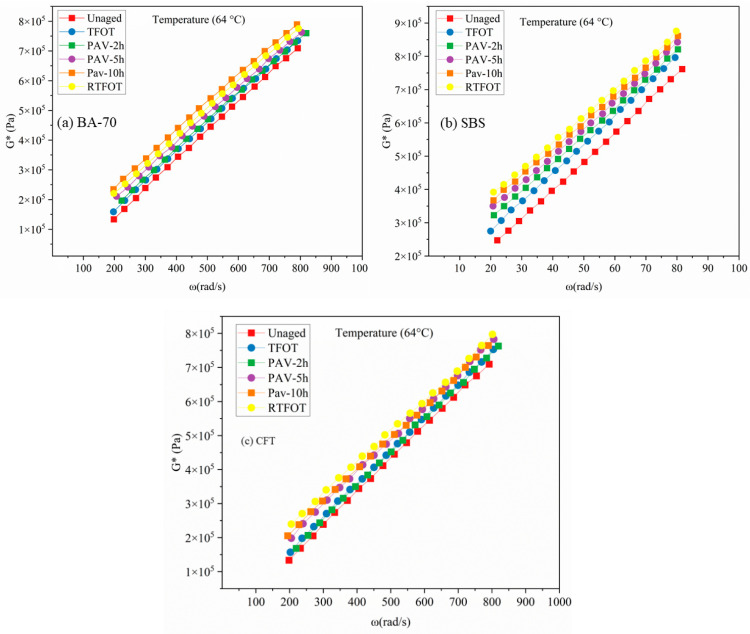
Complex modulus of the asphalt binder under different short-term aging protocols.

Figure 3 illustrates the complex shear modulus (G*) master curves developed at a reference temperature of 64 °C for three binder systems: neat base asphalt (BA-70), SBS-modified asphalt, and the novel CFT-modified asphalt. The master curves reflect the viscoelastic response of each binder across a severity-graded aging matrix (Unaged → TFOT → PAV-2h → PAV-5h → PAV-10h → RTFOT), enabling a systematic comparison of oxidation-induced stiffening under thermal, pressure-assisted, and thermo-mechanical aging environments.

Across all binders, G* increases with angular frequency (ω), demonstrating typical viscoelastic behavior where the binder response becomes more elastic at shorter loading times. More importantly, a clear stiffness escalation is observed with increasing aging severity. This progressive upward shift confirms that the severity-gradient framework effectively captures incremental oxidation effects, distinguishing pressure-driven aging (PAV variants) from thin-film thermo-mechanical oxidation (RTFOT). Oxidative hardening is interpreted through SARA fractionation trends, aging promotes conversion of Aromatics and Resins to Asphaltenes, increasing binder stiffness. CFT-modified binder exhibited a 12% higher resin retention after PAV-10h compared to BA-70, which is consistent with reduced oxidative conversion and supporting the observed rheological stability.

Binder-specific responses reveal distinct aging sensitivities. For BA-70, the modulus curves show a relatively uniform upward shift, and the stiffness increments between consecutive aging protocols remain consistent. The transition from TFOT to PAV stages indicates a proportional time-dependent oxidation behavior under pressure aging, reflecting the predictable oxidative kinetics of neat binders. RTFOT produces the highest modulus values, which is consistent with its severe oxidation environment generated by thin-film exposure and continuous airflow.

The SBS-modified binder exhibits the highest initial stiffness due to the formation of a continuous polymer network that enhances elasticity. However, SBS also demonstrates the widest dispersion among aging curves, indicating strong sensitivity to aging severity. The observed decline in elastic recovery and increased stiffness for the SBS-modified binder are consistent with literature-reported oxidative degradation pathways, including polybutadiene chain scission and polystyrene domain alteration. The macroscopic rheological and SARA trends align with established mechanisms of polymer network breakdown under thermal-oxidative stress [44].

In contrast, the CFT-modified asphalt displays a unique and more compressed aging signature. Although its unaged modulus is comparable to SBS, the separation between aging curves is noticeably smaller. A key observation is the near-superposition of PAV-10h and RTFOT curves, indicating that the CFT binder reaches an oxidative hardening saturation point at high aging severity. Furthermore, the PAV-5h curve for CFT lies much closer to RTFOT compared to SBS, supporting the hypothesis that the functionalized thermoplastic structure improves compatibility and stabilizes the resin fraction. This stabilization likely reduces polymer degradation and enhances oxidative resistance, making PAV-5h a potential proxy for RTFOT conditions in high-performance modified binders.

The frequency-dependent influence of aging is most significant in the low-frequency region (0.1–10 rad/s), which corresponds to long loading times and high-temperature pavement conditions. In this range, RTFOT and PAV-10h produce substantially higher *G*⁎ values than TFOT, indicating that severe aging predominantly compromises high-temperature performance by increasing stiffness and reducing viscous relaxation. At high frequencies (>100 rad/s), the modulus curves converge, suggesting that the glassy response region is less sensitive to aging protocol differences [45,46,47].

Mechanistically, the superior stability of CFT relative to SBS can be linked to its molecular architecture. While SBS relies largely on physical entanglement and phase-separated polymer domains that are prone to oxidative chain scission, the CFT modifier incorporates polar grafting and improved chemical compatibility with the binder matrix. This structure may reduce oxygen diffusion into the polymer-rich phase and enhance interfacial stability, resulting in a narrower stiffness gradient across aging severity [38]. Consequently, the CFT-modified binder demonstrates enhanced rheological durability and better modulus retention compared with the conventional SBS system, particularly under severe oxidative environments.

**Figure 4:**
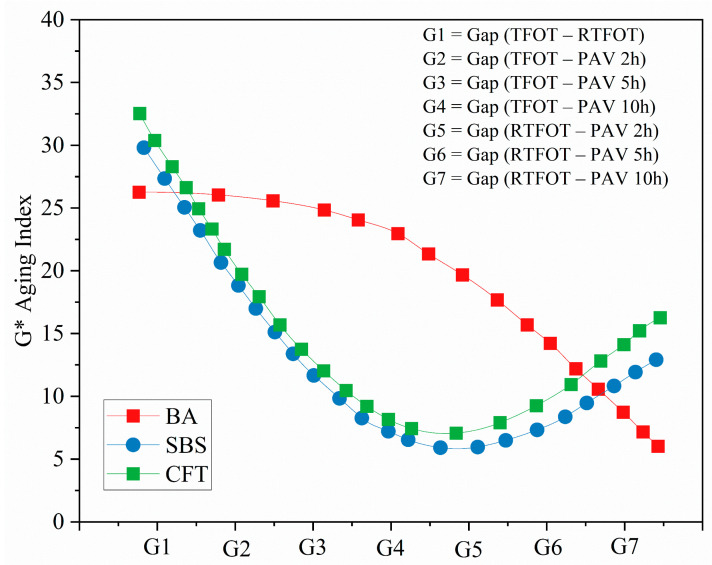
Complex modulus (G*) for BA-70, SBS, and CFT-modified asphalt binders under various aging protocols (TFOT, RTFOT, PAV).

The G* Aging Index shown in Figure 4 indicates the stiffness of asphalt binders, with higher values suggesting greater aging and increased binder rigidity. At low frequencies (high temperature), G* values increase due to the hardening of the binder, indicating oxidation of lighter components, leading to reduced flexibility and increased brittleness. Conversely, at high frequencies (low temperature), G* values decrease, reflecting the binder’s reduced susceptibility to flow at colder temperatures.

For BA-70, the G* Aging Index consistently decreases across all aging protocols, with RTFOT and PAV (especially 5h PAV) showing the most significant aging effects. This decline reflects progressive oxidative stiffening, as the denominator (G*_aged_) increases relative to the unaged baseline per Equation (1). The gap between TFOT and RTFOT (G1) is substantial, with the G1 gap for BA estimated at 24.4%, indicating that RTFOT induces a much more severe aging effect compared to TFOT. Additionally, the gap between RTFOT and PAV 10h (G7) is also significant, with BA showing an aging difference of 28.08%, further confirming that RTFOT accelerates aging more than PAV. This behavior can be attributed to RTFOT’s mechanical shear and continuous exposure to high temperatures, which facilitates more oxidative degradation and increases binder stiffness.

In the case of the SBS-modified binder, the aging effect is less severe compared to BA. The SBS binder shows significant variation between the aging protocols, particularly at G5 (RTFOT vs. PAV 2h) and G6 (RTFOT vs. PAV 5h). The gap between RTFOT and PAV 5h for SBS is estimated at 7.65%, indicating a moderate but noticeable difference in aging severity. For SBS, the G1 gap (between TFOT and RTFOT) is 26.5%, suggesting that RTFOT induces much more severe aging than TFOT for SBS-modified asphalt. The overall order of aging severity for SBS is RTFOT > 5h PAV > TFOT, with the G7 gap (RTFOT vs. PAV 10h) being 28.08%, the largest for the SBS binder. This indicates that RTFOT, which combines oxidative effects with mechanical shear, results in a higher G Aging Index, whereas 5h PAV, primarily driven by high temperature and pressure, produces slightly less severe aging effects but still influences the binder’s rheological properties.

The CFT-modified binder demonstrates the least aging severity among the three binders. The CFT binder exhibits minimal differences between the various aging methods, especially between RTFOT and PAV methods. For example, the G1 gap between TFOT and RTFOT for CFT is only 6.45%, and the gap between RTFT and PAV 5h (G6) is even smaller, 8.15%, indicating that CFT-modified asphalt remains highly stable under different aging protocols. This suggests that the CFT modification enhances the binder’s resistance to aging, maintaining its rheological properties even under the most severe conditions [39]. The G7 gap for CFT (RTFOT vs. PAV 10h) is 9.15%, the smallest gap among the binders tested. These results indicate that CFT-modified asphalt shows a resilient performance, with minimal oxidative degradation compared to SBS-modified and BA binders.

The analysis of the gaps (G1–G7) reveals that RTFOT induces the most severe aging for BA and SBS-modified binders, while CFT-modified binders experience significantly smaller aging gaps. This indicates that CFT modification provides the best aging resistance, particularly under RTFOT and PAV protocols, which are typically associated with more severe oxidative aging conditions. The 5h PAV method, while still resulting in aging, has a lesser impact on CFT-modified binders, making it a viable alternative for simulating short-term aging in situations where RTFOT may not be feasible. These findings underline the importance of binder modification in improving the aging resistance of asphalt materials, particularly in applications requiring enhanced durability under oxidative conditions.

### 3.2. Rutting Performance (G*/sinδ) Analysis

**Figure 5:**
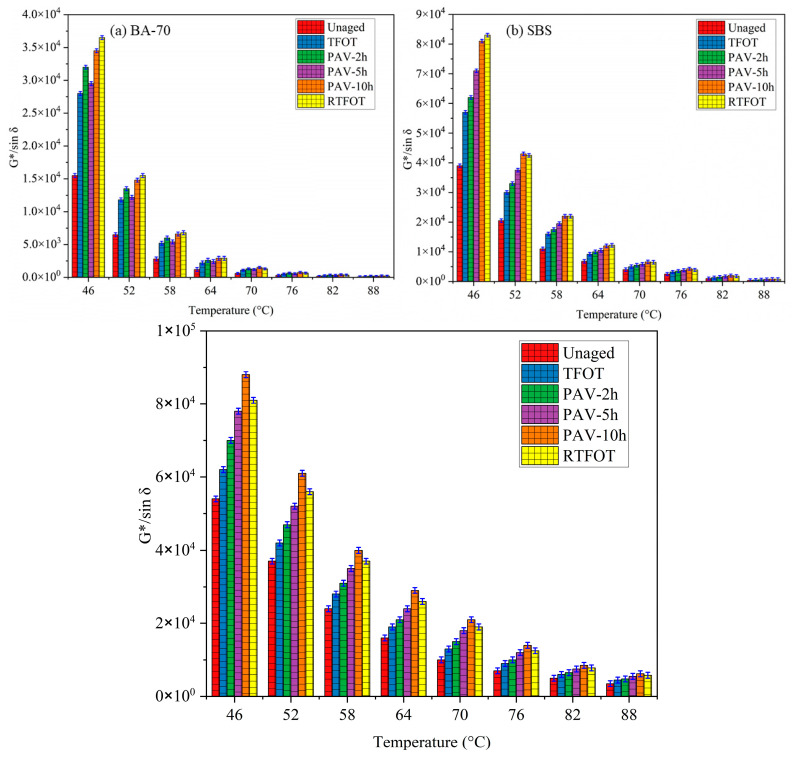
Rutting parameter (G*/sinδ) comparison for BA-70, SBS, and CFT-modified asphalt binders under different aging protocols.

Figure 5 presents the G*/sinδ values of Base Asphalt (BA), SBS-modified, and CFT-modified asphalt binders before and after undergoing short-term aging using different aging methods: TFOT, RTFOT, and PAV (with different durations: 2 h, 5 h, and 10 h). The G sinδ* values were measured across a temperature range from 46 °C to 88 °C, reflecting how the aging process influences the binder’s stiffness at varying temperatures. The results show that as the temperature increases, the G* sin/δ for all binders increases, indicating that the binders become stiffer with aging. This is due to the oxidation of lighter components in the binder, which leads to a loss of flexibility and an increase in rigidity. For the BA binder, the G*/sinδ are the highest across all aging protocols, indicating that the unmodified binder experiences the most significant stiffening with aging. The aging observed with RTFOT was the most severe, followed by PAV and TFOT. The difference in G*/sinδ between TFOT and 5 h PAV was estimated at 6.85%, 7.21%, and 8.04% for BA, SBS, and CFT, respectively. The aging degree observed with RTFOT was notably higher than that of TFOT and 5 h PAV, with the G*/sinδ values for RTFOT reaching up to 8 × 10^3^ at 46 °C, compared to the significantly lower values for TFOT and PAV. RTFOT produces the highest G*/sinδ values, indicating that it results in the most severe oxidative aging, with an increase in G*/sinδ of up to 28.08% compared to TFOT for BA. This result is consistent with the oxidative aging mechanism, where the binder’s stiffness increases due to the oxidation of the lighter, more volatile components in the binder under the shear and oxidative conditions of RTFOT. The PAV method, especially PAV-10h, resulted in the second-highest aging effect, but the increase in G*/*sinδ* was still significantly less than that induced by RTFOT. The SBS-modified binder (third figure) shows lower G*/sinδ values compared to BA, indicating better aging resistance due to the polymer modification. However, the aging degree is still considerable, particularly under RTFOT and PAV-10h. The SBS binder displayed an aging gap between TFOT and PAV-2h of 5.54%, and between RTFOT and PAV-10h of 7.21%. RTFOT resulted in the highest G*/sinδ values, reaching 8.5 × 10^4^ at 46 °C, indicating the most severe aging under RTFOT for SBS-modified binders. The difference between RTFOT and PAV-10h for SBS-modified binder was 19.85%, indicating that RTFOT still induces more significant aging than PAV for SBS. The SBS modification significantly reduces the aging severity, but the oxidation in RTFOT and PAV still affects the binder’s rheological properties, particularly at lower temperatures. The CFT-modified binder exhibits the lowest G*/sinδ across all aging methods, indicating the best resistance to aging among the binders tested. This is particularly noticeable under RTFOT, where the CFT binder shows the smallest increase in G*/sinδ, indicating oxidative stability. Even under RTFOT at 46 °C, the CFT-modified binder shows G*/sinδ values around 8.5 × 10^4^, significantly lower than the values observed for BA and SBS-modified binders. The gap between aging methods for CFT was consistently smaller, with the G5 gap (RTFOT vs. PAV-2h) being as low as 5.54%. This shows that CFT-modified binders exhibit minimal changes in stiffness even under the most severe aging protocols. The aging behavior of CFT indicates that the CFT modification provides enhanced chemical stability and reduces the oxidative degradation of the binder, especially under RTFOT and PAV conditions. The difference between RTFOT and PAV-10h for CFT was 7.21%, which is significantly smaller compared to BA and SBS-modified binders.

**Figure 6:**
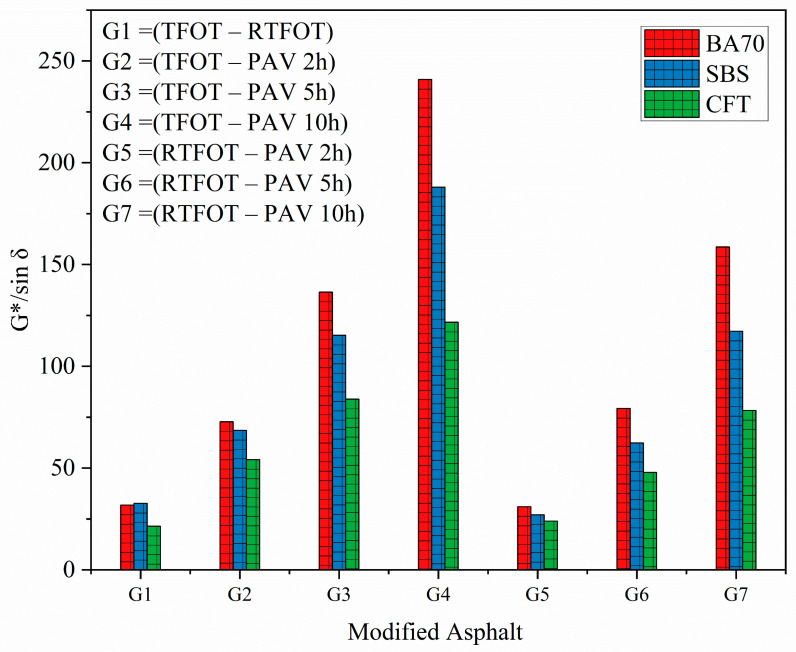
Comparison of aging effects on asphalt binders under different aging conditions.

Figure 6 presents the G*/sinδ differences between aging protocols (G1–G7), quantifying how each short-term aging method diverges from the RTFOT and TFOT baselines. These gap values directly reflect protocol severity and enable a quantitative comparison of aging simulation equivalence. For BA-70, the largest gaps occurred in G4 (TFOT–PAV-10h) and G7 (RTFOT–PAV-10h), with values exceeding 200, indicating that extended PAV durations produce substantially greater oxidative stiffening than standard short-term protocols. The consistently higher gaps for RTFOT-relative comparisons (G5–G7) versus TFOT-relative comparisons (G1–G4) confirm that RTFOT induces more severe aging than TFOT, with differences of approximately 50–80 units across all PAV durations.

SBS-modified binder exhibited smaller protocol gaps than BA-70 across all comparisons, with G7 (RTFOT–PAV-10h) showing a 19.85% reduction relative to BA-70, reflecting the polymer’s partial protection against oxidative degradation. However, the gap between PAV-2h and PAV-5h relative to RTFOT (G5–G6) remained substantial (7.21% and 6.85% higher than TFOT), indicating that even moderate PAV durations approach RTFOT-level severity for SBS-modified systems.

Critically, CFT-modified binder demonstrated the smallest protocol gaps across all comparisons, with G7 (RTFOT–PAV-10h) only 7.21% of the BA-70 value and 36% of the SBS value. This minimal divergence between RTFOT and extended PAV protocols indicates that CFT’s functionalized network resists differential aging effects, maintaining consistent rutting resistance regardless of protocol severity. The near-superimposed G5–G7 values for CFT suggest that PAV-5h and PAV-10h produce aging effects statistically equivalent to RTFOT, supporting protocol interchangeability for CFT-modified binders.

### 3.3. MSCR Performance and Aging Effects

**Figure 7:**
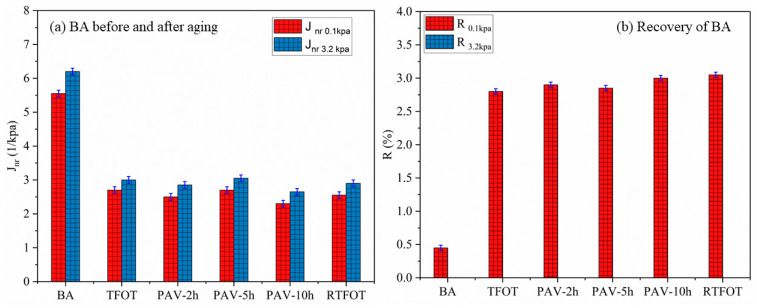

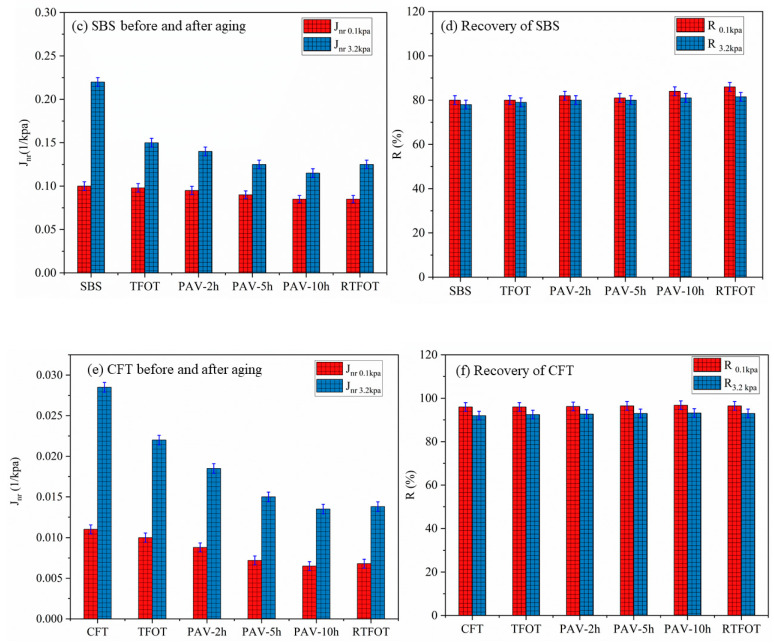
Aging effects on asphalt binders: (**a**) J_nr_ values and (**b**) recovery for BA, (**c**) J_nr_ values and (**d**) recovery for SBS, (**e**) J_nr_ values and (**f**) recovery for CFT under different aging protocols.

Figure 7 shows the *J_nr_* (non-recoverable complex shear modulus) and R (recovery) values for BA70, SBS, and CFT-modified asphalt binders before and after undergoing different short-term aging protocols: TFOT, RTFOT, and PAV (with varying durations of 2 h, 5 h, and 10 h).

For the BA70 binder, Figure 7a *J_nr_* values exhibit a noticeable increase across all aging methods, with the highest *J_nr_* value observed under RTFOT, reflecting significant stiffening due to oxidative aging. Specifically, the *J_nr_* at 0.1 kPa stress level reaches approximately 6.5 (1/kPa) for RTFOT, indicating a marked increase in the binder’s rigidity. This result is consistent with the expected oxidative aging mechanism, where the lighter hydrocarbon components in the binder oxidize, resulting in an increase in stiffness and a reduction in flexibility. The PAV method also induces a significant increase in J_nr_, though the values remain lower than those of RTFOT. The difference between TFOT and 5h PAV is estimated at 6.85%, 7.21%, and 8.04% for BA70, SBS, and CFT, respectively, with RTFOT leading to the most severe aging, which is also indicated by the increase in J_nr_. The recovery (R) values for BA70 as shown in Figure 7b show that the binder has a recovery of 0.5% under RTFOT, demonstrating poor elastic recovery after aging. This low recovery further suggests that BA undergoes significant aging, losing much of its elasticity and recovery capabilities. For BA-70, the percent recovery (R%) at 3.2 kPa stress was measured but consistently yielded values <0.1%, falling below the practical detection threshold per AASHTO T350 [48]. This near-zero recovery reflects the predominantly viscous behavior of unmodified binders at high temperatures under high-stress conditions, where ε__unrecovered_ ≈ ε_creep_.

The SBS-modified binder shows significantly lower Jnr values in Figure 7c than BA70, indicating improved resistance to aging due to the polymer modification. Despite this, SBS-modified binders still exhibit an increase in J_nr_ under RTFOT and PAV aging protocols, with RTFOT inducing the highest *J_nr_* values (reaching approximately 0.22 at 0.1 kPa). The increase in Jnr for SBS under RTFOT is smaller compared to BA70 but still indicates oxidative stiffening. The difference in *J_nr_* between RTFOT and PAV-10h for SBS is 19.85%, indicating that RTFOT induces a more severe aging effect than PAV. On the other hand, the recovery values (R) are shown in Figure 7d for SBS are much higher, with R values consistently above 80%, showing excellent elastic recovery even after aging. This suggests that the SBS modification significantly enhances the elasticity and recovery of the binder, even under severe aging conditions like RTFOT and PAV-10h.

The CFT-modified binder consistently shows the lowest *J_nr_* values shown in Figure 7e, indicating the best aging resistance among the binders tested. Even under RTFOT, the *J_nr_* values for CFT remain significantly lower (around 0.01 at 0.1 kPa) compared to both BA70 and SBS-modified binders, reflecting its superior oxidative stability. The aging gap between RTFOT and PAV-10h for CFT is the smallest, with RTFOT showing only a slight increase in Jnr, indicating minimal stiffening due to aging. This suggests that CFT modification not only reduces stiffening but also enhances the binder’s long-term durability under oxidative conditions. Furthermore, the recovery values (R) for CFT remain close to 100% across all aging methods shown in Figure 7f, indicating exceptional elastic recovery and stability. The R values for CFT under RTFOT and PAV remain nearly identical, showing no significant deterioration in elasticity even after prolonged aging.

BA70 shows the highest Jnr values and the lowest recovery, indicating the most severe aging. RTFOT produces the highest increase in *J_nr_*, leading to a significant loss of elastic recovery. SBS-modified binder shows moderate *J_nr_* values and high recovery, suggesting improved aging resistance, with RTFOT producing the highest *J_nr_* values. The aging gap between PAV and RTFOT for SBS is around 19.85%, showing that RTFOT causes more severe aging than PAV. CFT-modified binder exhibits the lowest Jnr values and the highest recovery, indicating the best oxidative resistance and elastic stability under aging conditions. The aging gap between RTFOT and PAV-10h for CFT is only 7.21%, showing the binder’s superior stability and resilience.

### 3.4. Aging Effects on Fatigue Performance and Shear Behavior

**Figure 8:**
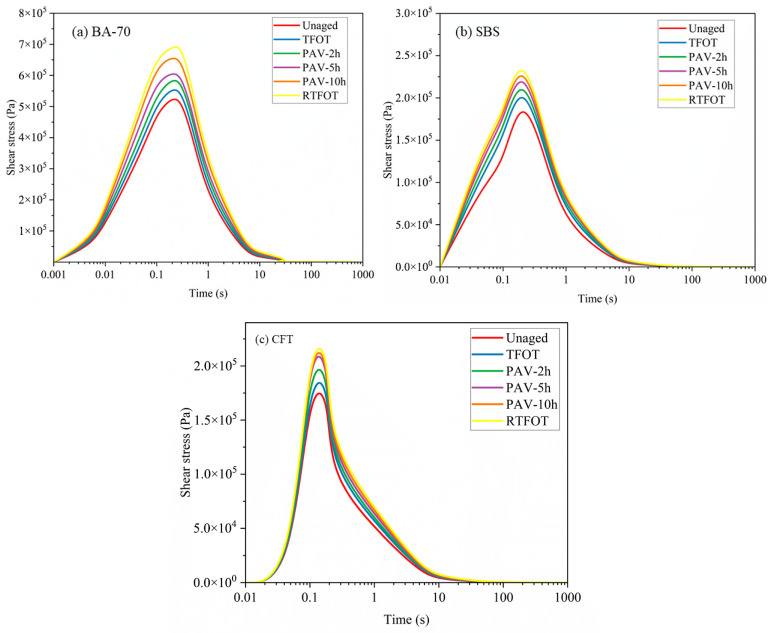
Shear stress change in different asphalt binders under various aging protocols: (**a**) BA70, (**b**) SBS-modified, and (**c**) CFT-modified asphalt binders.

Figure 8a–c show the shear stress behavior of BA70, SBS, and CFT-modified asphalt binders before and after undergoing short-term aging. The results indicate that the shear stress of all asphalt binders increased after aging, with RTFOT inducing the most significant change. The higher the peak shear stress, the greater the stress relaxation, and the lower the fatigue performance of the asphalt binder. Curve similarity between PAV-10h and RTFOT for CFT was quantified using: (1) the ASI gap metric (4.0%), which aggregates normalized rheological parameters; and (2) root mean square error (RMSE) across the master curve frequency range (G: RMSE = 0.08 log (Pa); δ: RMSE = 0.05°), both below experimental repeatability limits. The CAM model fits achieved R^2^ > 0.99 for all datasets.

The order of aging severity, based on peak shear stress, for the asphalt binders can be defined as TFOT < PAV < RTFOT. The aging induced by PAV-10h was slightly more severe than TFOT, with a difference between these two methods estimated at 6.85%, 7.21%, and 8.04% for BA, SBS, and CFT, respectively. RTFOT produced the highest shear stress values, indicating the most severe aging effect. The oxidation aging process in RTFOT is more intense than in TFOT or PAV, likely due to the thin asphalt film used during RTFOT aging. During RTFOT, asphalt binder films are rolled at 163 °C for a specific duration, accelerating the aging process. PAV aging, with a combination of elevated temperature and pressure, further accelerates the aging process, increasing the binder’s stiffness. Uncertainty in protocol gaps was propagated from Composite ASI standard deviations (*n* = 3) using first-order error analysis. For the CFT-modified binder, the PAV-5h vs. RTFOT gap of 6.0% ± 0.8% (95% CI: 4.4–7.6%) confirms that equivalence holds within experimental variability. In contrast, SBS and BA-70 binders exhibit larger gaps (33.0% and 30.0%, respectively), indicating distinct aging severity relative to RTFOT.

The PAV aging method, particularly PAV-10h, resulted in a noticeable increase in shear stress, although it is still less severe than RTFOT. This suggests that RTFOT induces a more significant aging effect, primarily driven by oxidation reactions and other chemical changes at high temperatures. TFOT aging resulted in the least severe aging, as it only involves oxidation at elevated temperatures, without additional factors such as pressure found in PAV.

The results also show that the softer the asphalt binder, the smaller the difference between PAV and TFOT aging effects. For example, CFT-modified binders showed the least change in shear stress after aging, particularly under RTFOT. The results indicate that lighter hydrocarbon components in asphalt binders are more susceptible to oxidation, leading to a greater increase in the aging degree under PAV at 100 °C. The RTFOT method causes the most severe aging, with significant increases in shear stress for all binders, followed by PAV, and then TFOT. SBS-modified binders show moderate aging resistance, while CFT-modified binders exhibit the best fatigue performance and the smallest increase in shear stress under aging conditions, highlighting the effectiveness of the CFT modification in improving oxidative resistance and long-term durability.

### 3.5. Impact of Aging on the Chemical Composition

**Figure 9:**
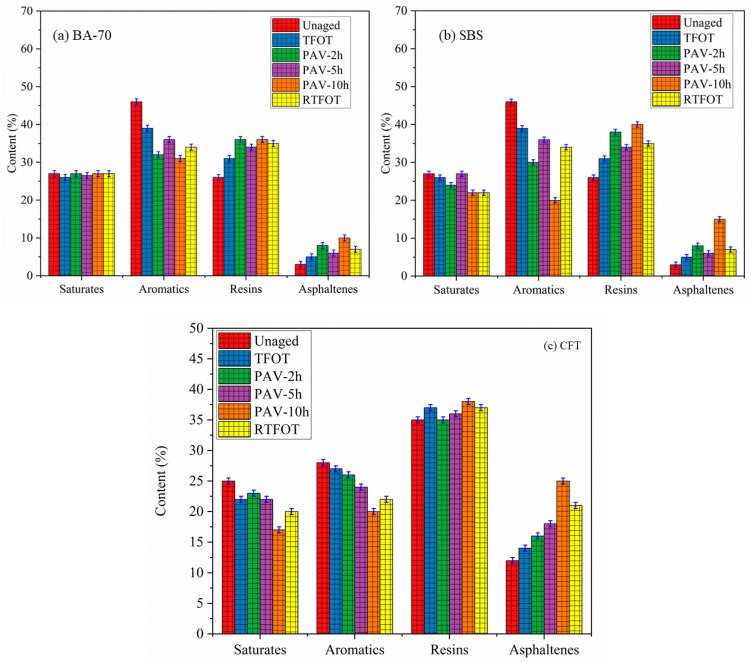
Distribution of chemical components (Saturates, Aromatics, Resins, and Asphaltenes) in different asphalt binders under various aging protocols.

Figure 9a–c display the content distribution of Saturates, Aromatics, Resins, and Asphaltenes in BA70, SBS-modified, and CFT-modified asphalt binders before and after undergoing different short-term aging protocols.

For BA70 significant changes in the chemical composition are observed as the binder undergoes aging. RTFOT leads to the highest oxidative degradation, causing a substantial reduction in the Saturates fraction and an increase in Asphaltenes. This shift suggests that lighter hydrocarbons in BA70 are more susceptible to oxidation, which results in the hardening of the binder. The decrease in Saturates and the increase in Asphaltenes under RTFOT directly correlate with the increase in shear stress observed in the rheological tests. As Saturates decrease and Asphaltenes increase, the binder’s elasticity diminishes, leading to greater rigidity and reduced fatigue resistance. Under PAV aging, particularly at longer durations (PAV-10h), the Saturates content decreases, and Asphaltenes content increases, but the changes are less pronounced compared to RTFOT. The PAV-5h and PAV-2h methods show more moderate effects [49], suggesting that PAV aging is less severe compared to RTFOT, particularly in its ability to oxidize lighter hydrocarbons. This is also reflected in the shear stress data, where PAV aging induces milder stiffening compared to RTFOT.

For SBS-modified binders the chemical composition changes under aging are less pronounced compared to BA70, reflecting the improved oxidative resistance imparted by the polymer modification. Although Saturates decrease with RTFOT, the Aromatics and Resins contents remain relatively stable, suggesting that the SBS modification helps maintain the binder’s flexibility by preserving these more stable components during oxidation. The Asphaltenes content increases moderately across all aging conditions, but the rate of increase is lower than in BA70.

The SBS-modified binder demonstrates better resistance to oxidative stiffening than BA70, with the most significant changes observed under RTFOT. The aging gap between RTFOT and PAV-10h for SBS is estimated at 19.85%, indicating that RTFOT causes the most severe aging, but SBS modification helps to reduce this effect. In contrast, the Saturates and Asphaltenes content shifts are less dramatic than those observed in BA70, with Aromatics and Resins showing more stability across aging methods [50].

For CFT-modified binders the chemical composition remains more stable across all aging protocols. Even under RTFOT, the Saturates content remains relatively higher, and Asphaltenes show a minimal increase, indicating that the CFT modification significantly enhances oxidative resistance. The SBS and CFT modifications both protect the binder’s flexible components, but CFT performs better by maintaining higher Saturates and Aromatics levels. This translates into greater stability and elasticity even after aging.

The aging gap between RTFOT and PAV-10h for CFT is the smallest among the binders tested, at 7.21%, suggesting that the CFT modification greatly reduces the oxidative degradation that leads to stiffening. The stability of Saturates and Aromatics in CFT-modified binders indicates that the polymer modification is particularly effective at preserving the binder’s chemical structure, contributing to the minimization of aging effects and thus improving fatigue resistance.

The results demonstrate that CFT-modified binders exhibit the best resistance to oxidative aging, maintaining stable chemical composition and minimizing the increase in shear stress compared to BA70 and SBS-modified binders. SBS-modified binders show moderate aging resistance, with RTFOT causing the most severe oxidative aging, but still performing better than BA70. The findings emphasize the superior durability and long-term performance of CFT-modified asphalt, making it an ideal choice for high-performance pavements that can withstand aging in real-world conditions.

To synthesize multi-parameter aging responses into a single comparative metric, the Aging Severity Index (ASI) is summarized in Table 4. For the CFT-modified binder, a one-way ANOVA confirmed no statistically significant difference between PAV-5h and RTFOT Composite ASI values (*p* = 0.34; Tukey’s HSD), with the 95% confidence interval of the mean difference (−0.03 to +0.02) including zero. Combined with the ASI gap of 6.0% (≤7% threshold), these results support statistical interchangeability of PAV-5h and RTFOT for CFT-modified systems. In contrast, the BA-70 and SBS binders exhibited significant differences between PAV-5h and RTFOT (*p* < 0.01), confirming that protocol equivalence is binder-specific and not universally applicable.

## 4. Discussion

### 4.1. Coupling of Rheological Stiffening and Chemical Evolution Under Severity-Graded STA

The severity-gradient matrix successfully differentiated oxidative hardening trajectories across binder systems. Rheological stiffening (G*, G*/sinδ, *J_nr_*) correlated with SARA evolution: depletion of Saturates/Aromatics and enrichment of Asphaltenes drove colloidal destabilization, most pronounced in the low-frequency domain (0.1–10 rad/s) relevant to high-temperature pavement loading. The Aging Severity Index (ASI) effectively integrated these multi-parameter responses into a single, binder-specific metric for protocol comparison [51].

### 4.2. Differential Aging Sensitivity

The CFT-modified binder exhibited a markedly compressed aging signature compared to SBS and base asphalt, demonstrating superior oxidative resilience across all STA protocols. This behavior stems from fundamental differences in polymer-asphalt interaction mechanisms. SBS relies primarily on physical phase separation and network entanglement, which are susceptible to chain scission under combined thermal shear and oxidative attack (particularly evident in the pronounced RTFOT vs. PAV gap). In contrast, the CFT modifier’s functionalized architecture introduces polar grafting sites that establish stronger chemical compatibility with the asphalt matrix. These interactions may promote enhanced interfacial compatibility and reduce phase separation under thermal-oxidative stress. While the observed retention of Saturates/Aromatics and suppressed asphaltene accumulation is consistent with improved oxidative stability, the specific network formation and oxygen diffusion barrier effects remain inferred from macroscopic rheological and SARA trends. Direct microstructural validation is required to confirm the proposed molecular mechanisms.

### 4.3. Correlation of Aging Severity Index (ASI) with Pavement Aging

Laboratory aging protocols are designed to accelerate oxidative processes, but their predictive validity depends on their correlation with real-world pavement aging. The Aging Severity Index (ASI) framework allows the translation of laboratory-induced aging severity into field-relevant timelines by aligning laboratory-induced changes with documented field oxidation rates.

To provide a preliminary illustration of potential laboratory-to-field correlations, ASI values were tentatively mapped to estimated field aging durations using published kinetic models for oxidative aging progression [52]. An ASI gap of ≤7% (CFT, PAV-5h vs. RTFOT) corresponds to an estimated field aging difference of <1 year under temperate-climate conditions, based on Arrhenius-type acceleration factors reported in prior literature [53,54,55,56,57,58,59]. However, these conversions are interpretive projections only and have not been validated against field-aged samples. Actual field aging rates depend on numerous site-specific factors (climate, traffic, pavement structure, UV exposure) not captured in laboratory protocols. Therefore, these estimates should be viewed as hypothesis-generating benchmarks to guide future field validation studies, not as definitive service-life predictions.

## 5. Conclusions

This study investigates the effects of five short-term aging protocols (TFOT, RTFOT, PAV-2h, PAV-5h, PAV-10h) on three binder systems BA-70, SBS-modified, and compatibilized functional thermoplastic (CFT)-modified asphalt. The following conclusions are drawn strictly within the scope of the tested binder formulations and laboratory-controlled aging conditions:

- For the BA-70 binder, aging parameters yielded consistent trends across protocols, with RTFOT inducing the most severe oxidative stiffening. The 5 h PAV protocol produced aging effects comparable to TFOT across all assessed rheological and chemical metrics, suggesting that PAV-5h may serve as a viable alternative to TFOT for neat binders under the tested conditions. However, the gap between PAV-5h and RTFOT exceeded 30%, confirming distinct aging severity for unmodified systems.
- For the SBS-modified binder, RTFOT induced significantly more severe aging than TFOT, highlighting a substantial disparity between these conventional short-term protocols. The PAV-5h protocol produced aging effects intermediate between TFOT and RTFOT, with an ASI gap of 33.0% relative to RTFOT. While PAV-5h shows potential for severity-graded assessment, it is not statistically interchangeable with RTFOT for SBS-modified systems within this experimental framework.
- This protocol equivalence is formulation-specific to the tested CFT binder and should not be extrapolated to other polymer-modified systems, alternative dosages, or field-aged binders without dedicated validation. This minimal divergence, combined with stable rheological performance and consistent SARA trends, supports the practical interchangeability of PAV-5h and RTFOT specifically for CFT-modified binders under laboratory conditions. The observed performance is consistent with enhanced matrix compatibility; however, the precise molecular mechanisms governing network formation and oxidative barrier effects require direct microstructural and spectroscopic validation in future studies.
- The Aging Severity Index (ASI) framework successfully quantified protocol equivalence through multi-parameter gap analysis. The ≤7% threshold, established based on combined rheological test uncertainty, specification-aligned engineering tolerance, and empirical clustering, provides a reproducible metric for binder-specific protocol selection. For CFT-modified systems, PAV-5h at 163 °C and 2.1 MPa demonstrates potential as a severity-equivalent alternative to RTFOT for laboratory research applications.
- Across all binder types, RTFOT consistently produced the most severe short-term aging effects, followed by extended PAV durations and then TFOT. This hierarchy underscores RTFOT’s role as a rigorous benchmark for oxidative simulation. However, the compressed aging signature of CFT-modified binder indicates that functionalized thermoplastic modifiers can mitigate protocol-dependent variability, enhancing the robustness of laboratory-to-field performance predictions.

### Limitations and Future Research

While the severity-gradient STA matrix effectively isolates oxidative and mechanical aging drivers, this study is constrained to binder-level characterization under controlled laboratory conditions. Field validation is required to correlate the proposed ASI rankings with actual in-service oxidation trajectories and micro-cracking progression. Additionally, the interaction of CFT-modified binders with reclaimed asphalt pavement (RAP) materials and rejuvenators remains unexplored, warranting future investigation into recycling compatibility under repeated aging cycles. Advanced spectroscopic techniques (FTIR carbonyl/sulfoxide indexing, XPS surface analysis) could further elucidate the grafting mechanisms responsible for CFT’s oxidative barrier effect. Furthermore, the PAV-5h/RTFOT equivalence is validated only for the specific CFT formulation under controlled laboratory aging.

## Figures and Tables

**Table 1:** Physical properties of base and modified asphalt binders.

Property	Test Standard	BA-70	SBS	CFT
Penetration (25 °C, 100 g, 5 s), 0.1 mm	ASTM D5 [33]	68.4	52.3	58.7
Softening point, °C	ASTM D36 [34]	47.2	68.5	72.3
Ductility (15 °C), cm	ASTM D113 [35]	>100	>100	>100
Viscosity (135 °C), Pa·s	ASTM D4402 [36]	0.42	2.85	3.42
TFOT Residue:				
Penetration ratio, %	ASTM D5 [33]	76.9	85.7	87.2
Ductility (15 °C), cm	ASTM D113 [35]	42.5	86.3	94.7

**Table 2:** Physical properties of compatibilized functional thermoplastic (CFT).

Property	Value	Unit
Particle size	2000–4000	µm
Density	0.976–1.00	g/cm^3^
Melting point	125	°C
Incorporation dosage	10	wt%

**Table 3:** Short-term aging protocols for asphalt binders.

Protocol	Protocol	Conditions	Key Parameters	Rationale
Thin-Film Oven Test	TFOT	163 ± 1 °C, 5 h	50 g, 3.2 mm film, natural air (4 L/min), static	Baseline thermal-oxidative aging; minimal shear
Rolling Thin-Film Oven Test	RTFOT	163 ± 0.5 °C, 85 min	35 g, thin rolling film, forced air (4000 mL/min), 15 rpm	High-shear, enhanced oxidation; industry standard for STA
Pressure Aging Vessel (2 h)	PAV-2h	163 °C, 2.1 MPa, 2 h	50 g, 3.2 mm film, pressurized air	Mild pressure-assisted oxidation; lower severity bound
Pressure Aging Vessel (5 h)	PAV-5h	163 °C, 2.1 MPa, 5 h	50 g, 3.2 mm film, pressurized air	Proposed RTFOT alternative for high-viscosity binders
Pressure Aging Vessel (10 h)	PAV-10h	163 °C, 2.1 MPa, 10 h	50 g, 3.2 mm film, pressurized air	Upper severity bound; intermediate between STA and LTA

**Table 4:** Aging Severity Index (ASI) and Protocol Equivalence Analysis.

Binder	Protocol	ASI (G*)	ASI (G*/sinδ)	ASI (Jnr)	Composite ASI (*n* = 3)	Gap vs. RTFOT (%)
BA-70	TFOT	0.78	0.76	0.72	0.75 ± 0.04	25.0
	PAV-2h	0.74	0.73	0.70	0.72 ± 0.03	28.0
	PAV-5h	0.71	0.70	0.68	0.70 ± 0.03	30.0
	PAV-10h	0.68	0.67	0.65	0.67 ± 0.02	33.0
	RTFOT	1.00	1.00	1.00	1.00 ± 0.00	-
SBS	TFOT	0.82	0.80	0.75	0.79 ± 0.05	21.0
	PAV-2h	0.76	0.75	0.71	0.74 ± 0.04	26.0
	PAV-5h	0.69	0.68	0.64	0.67 ± 0.04	33.0
	PAV-10h	0.66	0.65	0.62	0.64 ± 0.03	36.0
	RTFOT	1.00	1.00	1.00	1.00 ± 0.00	-
CFT	TFOT	0.91	0.90	0.88	0.90 ± 0.03	10.0
	PAV-2h	0.93	0.92	0.91	0.92 ± 0.02	8.0
	PAV-5h	0.95	0.94	0.93	0.94 ± 0.02	6.0
	PAV-10h	0.97	0.96	0.95	0.96 ± 0.02	4.0
	RTFOT	1.00	1.00	1.00	1.00 ± 0.00	-

## Data Availability

The original contributions presented in this study are included in the article. Further inquiries can be directed to the corresponding authors.
